# The Incidence, Management, and Outcome of Penetrating Bladder Injuries in Civilians Resultant from Armed Conflict in Baghdad 2005-2006

**DOI:** 10.1155/2009/275634

**Published:** 2009-04-05

**Authors:** Firas G. Petros, Richard A. Santucci, Naimet K. Al-Saigh

**Affiliations:** ^1^Al-Yarmouk Teaching Hospital, The College of Medicine, The University of Mustanisriya, Baghdad, Iraq; ^2^Detroit Medical Center, The Center for Urologic Reconstruction, Detroit, MI 48235, USA; ^3^Michigan State University College of Osteopathic Medicine, MI 48824-1316, USA

## Abstract

The purpose of this paper is to review the diagnosis, treatment, and outcomes of penetrating bladder injuries suffered by civilians in the Iraqi war zone. *Materials and Methods.* All civilian trauma cases received alive at Al-Yarmouk Teaching Hospital from January 2005 to August 2006 were reviewed for the presence of bladder injury. *Results.* 533 cases of penetrating abdominal trauma were identified, of which 177 (33%) involved the genitourinary (GU) system and 64 (12%) involved the bladder. Most (70%) were young males, and most (55%) had grade IV injuries. Associated injuries occurred in 63/64 (98%) of patients. 3 patients had missed bladder injuries, and all of these had complications related to their missed injury. Bladder-related complications occurred in 11% of cases, and mortality in 13%, all due to extravesical injuries. *Conclusions.* Penetrating bladder injury among civilians in Baghdad war zone resulted in 64 cases in 18 months. The initial detection rate is very high (98%), and after primary repair, lasting complications are rare. Morbidities from missed injuries were severe hematuria and vesicorectal fistula. However, (3%) of vesicorectal fistulae healed spontaneously with prolonged bladder drainage. Associated injuries are the rule in penetrating bladder injury patients, and must be diligently investigated and treated.

## 1. Introduction

The rate of wartime bladder injuries has stayed remarkably constant, at 15%–20%, over the last 60 years between World War II and the modern conflicts in the Balkans and the Gulf (1991) [1–3]. However, in the 21st century, near-universal use of body armor seems to have decreased the incidence of all genitourinary (GU) injuries in US soldiers to a very low percent 2% [4]. Civilians without body armor still have high rates of abdominal GU injuries and are though more susceptible than military personnel to bladder injuries. We have endeavored to describe our significant experience with 64 wartime bladder injuries among a civilian population in a war that torn Iraq during the years 2005-2006.

In peacetime, research and writings completed by civilian surgeons often educate the military surgeons as to the best practices. In wartime, the large numbers of the wounded allow military surgeons to advise civilian practitioners on improvements in the trauma field. It is in this spirit that we report these data, in order to improve peacetime and wartime understanding of the best care of penetrating bladder injuries which were obtained in peacetime.

## 2. Materials and Methods

From January 2005 to August 2006, all abdominal trauma cases received alive in the Emergency Department at Al-Yarmouk Hospital, which is one of the primary sites in Baghdad for acute management of civilian trauma, were reviewed for penetrating bladder injury.

 Injuries were staged using the American Association for the Surgery of Trauma (AAST) organ injury severity scale [5]. The mechanism of injury as well as the number and severity of associated injuries were noted.

All patients were managed in the Emergency Department with resuscitation, evaluation of bladder injury when Foley catheter was installed diagnosing hematuria. Sometimes those patients have frank wound(s) in the suprapubic areawhile others diagnosed during abdominal exploration and if time and clinical condition permitted, imaging studies were done as X-ray of pelvis occasionally cystogram. Urgent surgical exploration was performed in most cases since we were dealing with multiorgan-injured trauma victim patients with life-threatening concomitant injuries.

When bladder injury was encountered or looked for during laparotomy (if expected according to injury type and abdominal wound site), the principle was to repair the bladder with proper drainage. Through midline exploratory cystostomy, bladder walls, distal ureters, and bladder neck were all explored, avoiding as possible to interfere with any pelvic hematoma. Ureters were assessed by retrograde passage of ureteric catheters, in case of facing difficulties; exploration was done with repair or reimplantation according to the site of ureteric injury. Bladder injury site(s) was closed after limited debridement of any devitalized tissue with two layers of absorbable suture material. Difficult sites at bladder neck or trigone were managed in some cases with one-layer suturing from within.

In rectal involvement cases, debridement and selective separation of the organ walls were performed, to be sutured separately with interposition of omentum or any other available viable tissue if possible. We always tried to avoid overlapping suture lines, and together with colostomy, proper drainage of the area was done. All other injuries were dealt with accordingly. Transurethral Foley 2 way catheter of size 18–22 Fr was the routine way of postoperative drainage. Suprapubic cystostomy catheter was added in severely injured bladder where the repair was thought to be incomplete. Catheters were kept for 10–14 days according to injury assessment, and in some cases cystography preceded the removal especially for those with multiple bladder injuries and difficult bladder neck and/or trigone closure. Broad spectrum antibiotic cover was used to all patients starting from the Emergency Department. There was limited use of anticholinergic medications since in the majority of cases there was concomitant bowel injury.

SPSS version 11.5 (SPSS Inc., Chicago, Ill, USA) was used for data entry and analysis. Chi-square test of association was used whenever applicable. *P* value of less or equal to .05 was considered significant.

## 3. Results

**Figure 1:**
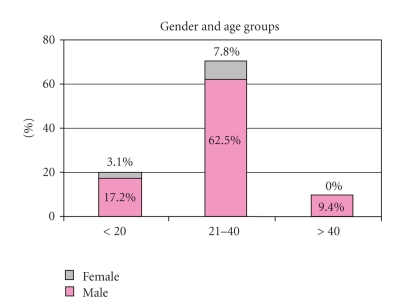
Age distribution of patients with penetrating bladder injury.

533 cases of penetrating abdominal trauma were identified, including 482 (89%) males and 51 (11%) females. Ages ranged from 4 to 60 years (median age 28) (Figure 1). 177 (33%) injuries involved the GU system and 64 (12%) involved the bladder. These 64 patients represented 36% of thepatients with GU injury.

**Figure 2:**
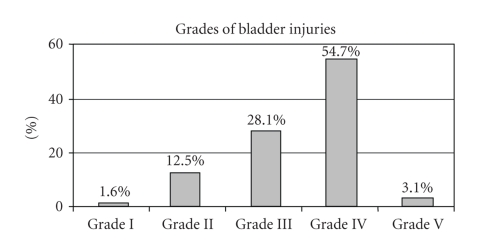
AAST organ injury severity scale grade of 64 penetrating bladder injuries.

**Figure 3:**
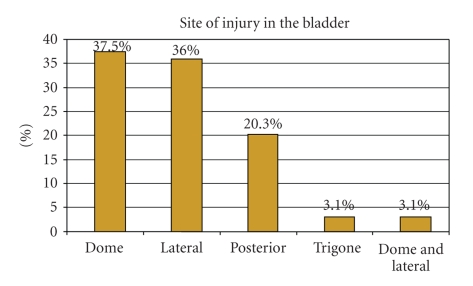
Location of 64 penetrating bladder injuries.

**Figure 4:**
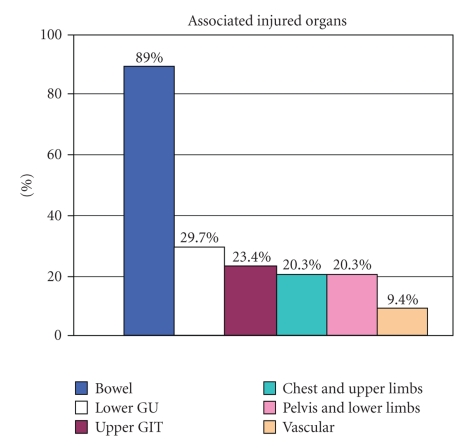
Concomitant injuries in 64 penetrating bladder injury patients.

All penetrating bladder injuries were due to bullets from pistols, rifles, and/or machine guns (78%), or shells from explosive devices (22%). No knife wounds were seen. 55% of the injuries were grade IV or higher (Figure 2) with equal involvement of the dome (38%) and lateral wall (36%) (Figure 3). Concomitant injuries occurred in all but 1 of the injured bladder patients (Figure 4).

Of the 64 bladder-injured patients, the bladder injury was identified and repairedsurgically during abdominal exploration in 60 (94%). Supravesical drainage was needed only in severe cases. Four cases (6%) were treated conservatively. Of these 4 patients, 3 had bladder injuries that were missed during surgical exploration. These 3 were grade III extraperitoneal injuries. The only case that was managed conservatively bychoice was a grade 1 injury proved by cystography and associated with lower limb injury.

Of the 64 bladder-injured patients, 49 (77%) suffered no significant morbidity. 8/64 (13%) died within 24 hours of injury, usually due to severe bleeding and grave-associated injuries, rather than from the bladder injury itself. Major vascular injuries were found in 5 (63%) of the patients who died.

**Figure 5:**
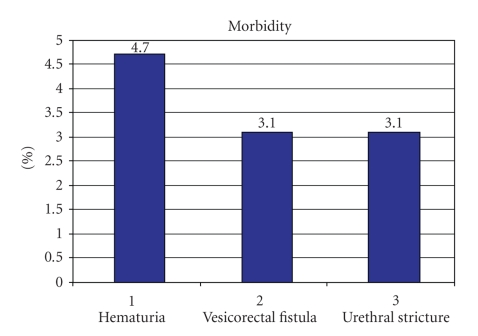
Complications in 64 patients with penetrating bladder injuries.

Serious complications occurred in 7 of 64 (11%) cases (Figure 5). Of these 7 cases, 3 had severe hematuria causing drainage problems and requiring blood transfusions; 2 had vesicorectal fistulae which healed with conservative management; 2 had urethral strictures, from concomitant urethral trauma. Of the 3 cases of missed bladder injury, 2 had severe hematuria and one had a vesicorectal fistula.

Mortality in this bladder-injured population was significantly associated with concomitant vascular (*P* = .0002) and chest (*P* = .003) injuries. Increased complications were related to those cases where injuries were missed compared to operatively repaired cases (*P* = .0001).

## 4. Discussion

Civil violence in Iraq has reached epidemic levels during the last 4 years, and increasing numbers of urological injuries are being seen among unprotected civilians. In this study, urologic injuries occurred in 33% (*n* = 177) of 533 penetrating abdominal injury patients, a high incidence that might be expected when dealing with civilians not wearing body armor. Of these patients with urologic injury, 36% had bladder injury, representing 12% of the entire population of patients with abdominal penetrating injury.

### 4.1. Comparison to Civilian and Wartime Series

The incidence of GU tract injuries reported in civilians is generally lower or comparable to that seen here. Civilian penetrating injuries to the bladder occurred in 11% of 155 victims with penetrating abdominal wounds seen in one US study [6], while during unrest in Belfast over a 10-year period, only 76 of such injuries were reported [7]. Some civilian series report bladder injuries in less than 5% of gunshot victims [6, 8].

Older series describing wartime bladder injury rates are also comparable to that seen in our civilian population here. In Vietnam and Croatia, bladder injuries occurred in 15% of those with GU injury [4, 8] and among 92 reported abdominal wounds in the Korean conflict; the bladder was involved in 11% of cases [8]. Bladder injury cases in Kuwait during the first Gulf War (1991) were 11% of all GU injuries among Iraqi wounded and 17% among US wounded [9, 10]. However, in modern conflicts in which body armor is universally used, the rate of bladder injuries is well below 2% [4].

The majority of the affected victims in our study were males (89%), reflecting the same male predominance in civilian penetrating abdominal injuries series (reported to be between 81% and 87%) [6, 10, 11].

In our study, 78% of the cases were caused by high velocity bullets, with only 22% from shells and explosives. Among US soldiers in the Iraq war, just the opposite was found, with 83% of the GU injuries due to fragmentation injuries and mines [9, 12].

### 4.2. Associated Injuries

In our series, all penetrating bladder cases were associated with injuries to other organs, which coincide with result reported widely in literature [4–6, 8–11, 13]. Bullets caused injury to a greater number of intra-abdominal organs compared to explosive fragment wounds (2.05 versus 1.56 organs per patient), which also coincided with what has been reported in literature [14].

Bladder injuries commonly co-occur with colon/rectal injuries. In one series, bladder injuries occurred in 13% of penetrating rectal injuries [15], and in another, 1/3 of patients with extraperitoneal rectosigmoid gunshot wounds had bladder involvement [10]. In our series, the colon was injured in 33% and the rectum injured in 22% of the bladder trauma cases. In general, they were successfully managed, as no significant increase in mortality was found. In this study and others, small and large bowels were also commonly injured, with rates reaching 34% and 33% for small and large bowels, respectively [6, 8, 9]. Urethral injury was found in 3% of the cases.

Major vascular injuries accompanied 9% of our bladder cases. It was found to be statistically a significant cause of death in 63% of the dead cases. This factor represents the major impact on mortality rates in different studies [6, 16].

We had ureteric involvement in 4 (6%) cases, while it is usually found in 4%–6% of all urological injuries [9]. Three of our cases were discovered during surgery and treated with immediate reimplantation, while the fourth one was missed, and reimplantation was done successfully later.

### 4.3. Surgical Technique

Our surgical technique reflects what is routinely done in many studies [5, 6, 9, 10, 13, 17], except that we used only suprapubic drainage in more severe and massive bladder injuries, and no damage control principles were required. Complications occurred in 7 out of the 64 bladder injuries (11%), representing 5% of the surgically repaired cases, and appeared lower than rates described elsewhere which can range as high as 33% [6, 9]. Complications occurred in all 4 missed and conservatively treated injuries. Of the 3 cases of missed bladder injury, 2 had severe hematuria and one had a vesicorectal fistula while the only case of conservative management was accompanied by severe hematuria. Higher complication rates in cases treated conservatively have been described previously [5]. The complication of hematuria and rectal fistula healed spontaneously, and this coincides with other studies [5, 6, 9, 10].

One of the vesicorectal fistulae resulted from partial dehiscence of repair of concomitant bladder and rectal injuries which was evident clinically and radiographically at end of the first postoperative week, while the other one resulted from missed bladder and rectal injuries diagnosed at the fifth postoperative day by the same way. Healing of vesicorectal fistula was achieved by prolonged bladder drainage by transurethral catheterization formore than 2 weeks (up to 1 month) depending on the size of the fistula, nothing by mouth, IV fluid; antibiotic cover and follow up cystography.

We did not see in our study chronic complications like bladder neck stenosis, erectile dysfunction, and overactive bladder symptoms such as what have been described elsewhere and can involve between 5% and 21% of cases [5].

Mortality rate after isolated bladder injury has plummeted over the last century. Mortality rates dropped from 35% in the 1930s [11] to 16% during 1940s [6], then to 10% in the Korean War [8], reaching 1.3% in civilian abdominal injuries in the sixties [6]. However, mortality rate in bladder injuries patients with other associated severe injuries can still reach high levels between 12% and 22% [5, 18]. This reflects the severity of the associated injuries rather than the bladder injury itself. Most of this mortality is due to vascular injury, and death rates are reportedly much higher in those with longer evacuation times, reflecting the risk of exsanguinations after these types of injury [4, 7–9, 16, 18].

In our study, mortality rate was 13%, death occurred in all cases within 24 hours of injury, and major vascular injuries were found in 63% of these victims. Both vascular and associated chest injuries were statistically associated with higher mortality rate in our series. The grade of bladder injury was not found statistically related to mortality rate, reflecting the fact that death is related to other organs injured rather than the bladder itself. Bullets were responsible for 75% of injuries, indicating the higher incidence of major vascular injuries with bullet wounds (20% versus 9%) which ultimately ended with greater mortality rates (7% versus 2%) [14].

## 5. Conclusion

The incidence of urological injury is high in unprotected civilian victims of wartime injuries in modern Baghdad. The majority of penetrating bladder injuries were associated with concomitant organ injury, and severe blood loss and death were not uncommon. Management of bladder or indeed any GU injury should emphasize investigation for concomitant injury.

Complications are generally uncommon, and most often resolve spontaneously. Even some severe complications such as vesicorectal fistula heal with prolonged urinary drainage. Missed injuries have a higher complication rate, so it is important to accurately screen for bladder injuries in a penetrating trauma population. Suprapubic tubes are occasionally required after primary bladder repair of severe and massive bladder injury. Wartime series such as this can help to advise civilian trauma surgeons as to the best practices through the vast experience when dealing with such multiorgan-injured patients especially in the unique challenges of Iraq's situation in which there is deficiency of experienced trauma surgeons, and a lot of the difficult work is dealt with by junior residents, in addition to deficiency in equipments. Yet, one can only salute the great job which is done every day, by unknown soldiers; those are the Iraqi doctors and medical personals on the line of fire in Baghdad hospitals.
